# Report on a Milking Mule: Milk Qualitative Characteristics during Lactation

**DOI:** 10.3390/ani14111585

**Published:** 2024-05-27

**Authors:** Mina Martini, Andrea Degl’Innocenti, Iolanda Altomonte, Irene Sodi, Carlotta Bocci, Diana Fanelli, Rebecca Moroni, Duccio Panzani, Francesco Camillo, Federica Salari

**Affiliations:** 1 Dipartimento di Scienze Veterinarie, Università di Pisa, 56124 Pisa, Italy; mina.martini@unipi.it (M.M.); irene.sodi@phd.unipi.it (I.S.); c.bocci88@gmail.com (C.B.); diana.fanelli@unipi.it (D.F.); rebecca.moroni@phd.unipi.it (R.M.); duccio.panzani@unipi.it (D.P.); francesco.camillo@unipi.it (F.C.); federica.salari@unipi.it (F.S.); 2 Dipartimento di Biotecnologie Mediche, Università di Siena, Policlinico S. Maria alle Scotte, 53100 Siena, Italy; andrea.deglinnocenti@unisi.it

**Keywords:** equid milk, mule milk, milk quality, minerals, fatty acids

## Abstract

**Simple Summary:**

Mules cannot reproduce naturally; however; they can host embryos, gestate normally, and act as surrogate dams, producing milk for the foal. There are only a few studies on the composition of mule milk. We evaluated the chemical quality of the milk obtained from a mule dam that had foaled after receiving a mule embryo transfer. The quality of the mule milk was similar to that of horse and donkey milk. Monounsaturated and polyunsaturated fatty acids made up half of the total fatty acid content. Linoleic acid and linolenic acid were the main polyunsaturated fatty acids in the milk. The milk composition changed throughout lactation. Protein, fat, and ash decreased significantly from early lactation. The n3 polyunsaturated fatty acids decreased at the end of lactation. The changes in milk composition throughout lactation are probably due to adaptations to the growth requirements of the foal.

**Abstract:**

Despite their inability to reproduce naturally, mules can host embryos and be surrogate dams. The aim of this investigation was to increase our knowledge of the qualitative composition of mule’s milk and its variations throughout the whole lactation period—namely, from 6 h after foaling to 180 days in milk (DIM). Milk was obtained from a mule dam that had foaled after receiving a mule embryo transfer. For each sample, the gross, mineral, and fatty acid composition was evaluated. The average quality of the mule milk was as follows: protein 1.97 g 100 mL^−1^, fat 0.90 g 100 mL^−1^, and ash 0.39 g 100 mL^−1^. Saturated fatty acids made up, on average, 50.00 g 100 g^−1^ of fat. Monounsaturated and polyunsaturated fatty acids made up half of the total fatty acid content (31.80 g 100 g^−1^ and 18.2 g 100 g^−1^ of fat, respectively). Linoleic acid and linolenic acid were the main polyunsaturated fatty acids in the milk. The milk composition changed throughout lactation. Dry matter, protein, fat, and ash decreased significantly from early lactation (6 h to 14 DIM). The n3 polyunsaturated fatty acids decreased at the end of lactation. The changes in milk composition throughout lactation are probably due to adaptations to the growth requirements of the foal.

## 1. Introduction

Equids are a relatively homogeneous group of moderately large grazers adapted to semi-arid conditions. There are many different equine hybrid combinations, such as those crossbred between Przewalski (*Equus prezwalskii*, 2n = 66) and domestic horses (*E. caballus*, 2n = 64), wild (e.g., *E. hemionus*; 2n = 54) and domestic (*E. asinus*; 2n = 62) asses, and between various subspecies of zebras. Mules (jackass × mare, 2n = 63) are the most common equine hybrids from *E. caballus*, (2n = 64) and *E. asinus*, (2n = 62) [1]. Hinnies (horse stallion × jenny, 2n = 63) seem to be much less common and diffused. In fact, when natural breeding between a horse stallion and a jenny occurs, significantly lower conception rates are achieved [2].

The effect of hybridization on an organism is variable. Particular traits may either be determined by one parent, be intermediate between the parental traits, be inferior to the traits of both parents, or be superior to both. Regarding morphological traits, mules inherit the best from both parents: they are stronger, carry more weight than horses of the same size, and have the sure-footedness of the donkey [3]. These characteristics made mules important as working animals for agriculture and during the First World War [4]. Today, mules are once again valued, and donkeys and mules continue to have a positive impact in developing countries; their economic value stems from work, transportation, packing, and tourism [1].

Albeit rarely capable of conceiving naturally, mules can host embryos and gestate normally [5]. Equine embryo transfer enables several foals to be obtained from a mare each year [6], which is also a valuable strategy for preserving endangered equid species [7]. The usefulness of cycling mules as recipients of horse [5] and donkey [8] embryos has been demonstrated. The possibility of a foal being born after the transfer of a donkey embryo to a non-cycling mule has also been reported [9].

Mules can be surrogate dams and produce milk for the foal [5]. Milk provides the foal with all the energy and nutrients needed during the first few weeks post partum, and it remains the primary nutrient source until weaning. While information on the milk composition of commercially exploited animals is available, as well as that of donkeys and horses, very little information is available on the milk composition of other equids [10]. 

To the best of our knowledge, there are no studies on the composition of mule milk, except for some are scattered and fragmentary data [11]. Milks from different species differ greatly in composition; however, some authors have speculated that the milk from species in the same taxonomic order tends to have a similar composition [11].

The aim of this preliminary investigation was to increase our knowledge on the qualitive composition of mule milk and its variations throughout the lactation period.

## 2. Materials and Methods

Milk was obtained from a twelve-year-old tobiano female mule that had foaled after receiving a mule embryo transfer at the Department of Veterinary Science of the University of Pisa. The mule was stabled at the VTH of Pisa University and was routinely checked by veterinarians to exclude clinical and reproductive abnormalities.

Milk samplings were performed at 6, 12, and 24 h after foaling, after which they were performed at 2, 3, 4, 5, 6, 7, 10, 13, 17, 20, 24, 27, 30, 37, 45, 52, 60, 90, 120, 150, and 180 days after foaling, or days in milk (DIM). The foal was separated from the mother for 3 h before milking, with the exception of the first two samplings (6 h and 12 h after delivery), when the foal was separated for 30 min before milking. The separation was carried out using a fence that enabled the animals to have tactile and visual contact, but which prevented the foal from suckling. In addition, at each sampling, the foal was kept close to the mare to promote oxytocin release, and the udder was completely emptied via manual milking. A total of 24 milk samples were collected and stored at −20 °C.

All samples were analysed in duplicate for dry matter, protein, fat, and ash content using the Association of Official Analytical Chemists methods [12]. Mineral analysis was carried out after digestion with nitric acid and perchloric acid, and calcium, magnesium, potassium, and zinc were quantified using an atomic absorption spectrophotometer (AA-7000, Shimadzu, Kyoto, Japan), while phosphorous was quantified using a colorimetric method [12] with a UV-Vis spectrophotometer (V530, Jasco Inc., Easton, MD, USA). For the fatty acid analysis, milk fat was extracted following the Roese–Gottlieb method [12]. Methyl esters of fatty acids were prepared according to Christie, as described in the AOAC methods [12]. A gas chromatograph (Clarus 480, PerkinElmer, Norwalk, CT, USA), equipped with a flame ionization detector and a capillary column (ThermoScientific TR-FAME 60 m × 0.25 mm ID; film thickness 0.25 μm, Fisher Scientific, Loughborough, Leicestershire, UK) was used for the fatty acid analysis.

The gas chromatographic conditions and oven program were as described in our previous work [13]. The peak areas of individual fatty acids were identified using a fatty acid standard injection (Food Industry FAME Mix—Restek Corporation, Bellefonte, PA, USA) and quantified as the percentage of total fatty acids. 

### Statistical Analysis

In order to detect changes in the chemical composition of the milk secretion, obtained data were grouped into five periods on the basis of DIM:

- Phase I of lactation: from 6 to 24 h postpartum;
- Phase II of lactation: from 2 to 4 DIM;
- Phase III of lactation: from 5 to 14 DIM;
- Phase IV of lactation: from 17 to 90 DIM;
- Phase V of lactation: from 90 to 180 DIM.

Data were tested for normal distribution and homogeneity of variances using a Shapiro–Wilk and Levene tests. They were analysed via ANOVA, using JMP software version 5 [14], considering the lactation phase as the fixed effect. Tukey test was used for multiple comparison. Significant differences were considered at *p* ≤ 0.05.

## 3. Results and Discussion

The average composition of the mule milk is shown in Table 1. 

Comparing the chemical composition with the few parameters evaluated in mules by Schryver et al. [15], our findings show a slightly higher dry matter (10.84 vs. 10.1 g 100 g mL^−1^ of dry matter) and lower ash (0.39 vs. 0.48 g 100 mL^−1^ of ash) content. 

The milk protein, fat, and ash percentages were intermediate to the average content reported for donkey milk (1.85, 0.70, and 0.35 g 100 mL^−1^, respectively) [16] and for mare milk (2.14, 1.21, and 0.42 g 100 mL^−1^, respectively) [17].

The main milk mineral was calcium, followed by potassium and phosphorous, as also reported for horse and donkey milk [16,17]. According to some studies on mares, equid milk provides a sufficient amount of potassium, magnesium, and zinc for the foal’s growth, but only 78% of calcium requirements and 90% of phosphorous requirements. Nursing foals should therefore have early access to good-quality forage or creep feed to correct for milk deficits [18].

Calcium, potassium, phosphorous, magnesium, and zinc were within the wide range reported for equids [16,17] (calcium: jenny 360–1180 mg L^−1^; mare 500–1350 mg L^−1^; phosphorus: jenny 320–840 mg L^−1^, mare 200–1210; potassium: mare 250–800 mg L^−1^; jenny 204–969 mg L^−1^; magnesium: mare 40–110 mg L^−1^; jenny 20–110 mg L^−1^; zinc: mare 0.9–6.4 mg L^−1^; jenny 2.16–4.56 mg L^−1^). 

To the best of our knowledge, this is the first report on the fatty acid composition of mule milk. The average saturated fatty acid (SFA) values (Table 1) (50.41 g 100 g^−1^ of fat) were intermediate between those reported for donkey milk (52 g 100 g^−1^ of fat) [16] and horse milk (47.40 g 100 g^−1^ of fat) [17].

In addition, the monounsaturated fatty acid (MUFA) values (31.79 g 100 g^−1^ of fat) were slightly higher than the average content of donkey milk (23.00 g 100 g^−1^ of fat) [19,20], and more similar to horse milk (31.14 g 100 g^−1^ of fat) [21,22]. On the other hand, the polyunsaturated fatty acid (PUFA) values (18 g 100 g^−1^ of fat) were intermediate between the findings for donkey milk (14.19 g 100 g^−1^ of fat) [19] and horse milk (22.01 g 100 g^−1^ of fat) [21,22]. 

Among all the SFAs (Table 1), palmitic acid (C16:0) was the most represented (22.24 g 100 g^−1^ of fat), which is very similar to donkeys (22.5 g 100 g^−1^ of fat) [23,24] and horses (22.74 g 100 g^−1^ of fat) [21,22]. 

Although often considered to have adverse effects on chronic disease in humans, C16:0 is an essential component of tissue lipids, acting in the secretion and transport of lipids and in the formation of signal molecules [25].

The other most represented SFAs were C10:0 (7.55 g 100 g^−1^ of fat) and C14:0 (6.92 g 100 g^−1^ of fat). C10:0 was closer to the average values reported for donkey milk (8 g 100 g^−1^ of fat) than to those for horse milk (6.67 g 100 g^−1^ of fat). The C14:0 content was in agreement with the literature data on horse milk (6.37) [21,22], while being higher than the range described for donkeys (5 g 100 g^−1^) [23,24]. The relatively low levels of stearic acid (C18:0) in equid milk can be explained by dietary factors and the low Δ-9 desaturase activity in the equine mammary gland [26], which catalyses the formation of MUFAs from stearic and palmitic acids. In fact, the most represented unsaturated fatty acid was oleic acid (C18:1 c9) (23.69 g 100 g^−1^ of fat), which was intermediate between donkey milk (19.5 g 100 g^−1^ of fat) [16] and horse milk (25.15 g 100 g^−1^ of fat) [17].

Conjugated linoleic acid (CLA) was present in very small amounts (0.04% of fatty acids) in mule milk, in agreement with the literature on horse and donkey milk [22,24]. The difference between the CLA level in ruminant and equine milk is due to the lack of biohydrogenation of C18:2 n6 before absorption in the small intestine and a very limited absorption of both CLA and *trans*-vaccenic acid from the large intestine [26].

The most represented PUFAs were linoleic acid (C18:2 n6, LA) and linolenic acid (C18:3, ALA), which are precursors of n6 and n3 fatty acids, respectively. The values of C18:2 n6 and C18:3 n3 were 9.53 and 7.80 g 100 g^−1^ of fat, respectively. The average content of LA was lower than values found in donkey (13 g 100 g^−1^ of fat) and horse milk (14.94 g 100 g^−1^ of fat) [16,19,21,22], while ALA was intermediate between donkey (8 g 100 g^−1^ of fat) [27] and horse milk (7.05 g 100 g^−1^ of fat) [22]. 

In the literature [21,22,27], there are fairly wide ranges of LA and ALA content for donkey and mare milk (LA 9–17 g 100 g^−1^ of fat for donkeys; 6–16 g 100 g^−1^ of fat for horses, ALA 2–14 g 100 g^−1^ of fat for donkeys; 5–22 g 100 g^−1^ of fat for horses), which are probably due to differences in the fatty acid composition of diets. Since PUFAs consumed by equids are not microbiologically dehydrogenated prior to intestinal absorption, the milk long-chain fatty acid composition is related to the fatty acid profile of feedstuffs [26,28]. Diets richer in forage have more ALA than those rich in concentrates.

C20:5 (eicosapentaenoic acid, EPA) and C22:6 (docosahexaenoic acid, DHA) were present in low quantities in milk (both 0.02 g 100 g^−1^ of fat). They are not considered as essential fatty acids for foals, because they can be synthesized to some degree from the essential ALA [18]. n-3 FA has immune-modulating effects in a number of species, potentially affecting the humoral immune system. The beneficial effects for foals, if any, remain to be determined [29].

Regarding changes in the gross composition of milk during lactation (Table 2), higher contents of dry matter, protein, fat, and ash were found in the first lactation phases (phases I–III).

Similarly, in mare milk, dry matter has been found to be higher in colostrum (6 h after parturition) [22]. As has also been reported in other studies on equids, colostrum and transition milk are also richer in protein, fat, and ash [23,30]. The abundance of nutrients at early lactation is important to support the nutritional needs of the neonatal foal, given the rapid growth and development at this stage. In fact, during the first day of life, a foal has a high metabolic rate and poor reserves in the form of glycogen or fat, and it is thus important to provide it with nutrients and energy in order for it to thermoregulate [18]. 

During lactation (Table 2), dry matter, protein, and ash decreased progressively and significantly (*p* < 0.01) from the IV phase, while fat (*p* < 0.01) decreased in the IV phase, remaining stable thereafter. The gradual reduction in protein during lactation was in agreement with the reports on donkeys [31,32] and on mares [21,22].

The variations in the milk composition throughout lactation could be due to physiological adjustments to the nutritional requirement of foals.

Regarding trends in milk fat during lactation, the literature on equid milk has reported conflicting results, with scholars either not finding changes [33,34] or detecting an increasing percentage of fat in donkey milk [22,35], and other authors describing decreasing trends in mare milk [21,28]. The conflicting results in the literature may be due to the collection method, to the particular anatomical and physiological features of equids, and to the difficulty in completely emptying the udder. This is directly related to the fat content of milk, since the residual fraction milk is rich in fat [33].

Very little information is available on the mineral composition of milk from female mules during lactation. Our findings on phosphorus and potassium (Table 2) in the first three phases of lactation are slightly higher than those (530 mg L^−1^ P; 845 mg L^−1^ K; 1.1 mg L^−1^ Zn) in the only paper available, by Schryve et al. [11], who analysed the mineral composition in early lactation from a mule mare, whereas magnesium was slightly lower (144–110 vs. 150 mg L^−1^ Mg).

As previously reported for donkey milk [23], the magnesium content of colostrum during the first 6 h after foaling was 2–3 times greater than that observed in the subsequent lactation period. Colostrum contributed to the higher concentration of magnesium and potassium in the first phase of lactation. In fact, colostrum is known to be rich in these components, which also seems to facilitate the expulsion of meconium [23]. Magnesium decreased progressively and significantly from the first to the fifth phase of lactation. Potassium was higher in the first three phases of lactation, as observed in donkey colostrum [23], and decreased (*p* < 0.01) by approximately one half in the IV phase. 

The decreasing trends observed during lactation for phosphorus, magnesium, and potassium are consistent with the decline in ash content and have also been observed both in donkey and mare milk [31,36]. Over time, the content of zinc did not change significantly. 

In addition, calcium showed minimum values at the beginning of lactation (phase I), which has also been reported in previous studies on the first days of lactation in donkeys [23]. Calcium showed a maximum value in the III phase of lactation and significantly decreased thereafter.

Overall, the content of SFAs, MUFAs, and PUFAs (Table 3) showed no significant differences during lactation, in agreement with previous studies on donkey milk [23,24]. However, in mares, some authors have found high concentrations of SFAs in colostrum, compared to mature milk [22].

Regarding short-chain fatty acids, C4:0 increased significantly in phases II and III of lactation, in agreement with findings by Pikul et al. [21] and Barreto et al. [22] for mare milk, and therefore decreased in phase IV (after 17 DIM). C6:0 progressively increased and then decreased in the last lactation phase, as already observed in horses [21,22]. C10:0 showed a decreasing trend.

C15:0 and C15:1 progressively increased through lactation. Similar trends for C15:1 have been reported in mare milk [21].

C14:1, C16:0, and C17:0 presented similar trends with maximum values in phase II (from 2 to 4 DIM) and phase V (from 90 to 180 DIM); similar trends for C14:1 have been reported by Barreto et al. [22].

On the other hand, C18:0 showed a peak in phase III (from 5 to 13 DIM), and tended to decrease at the end of lactation, as also reported by Pikul et al. [21] and Barreto et al. [22] in mare milk. 

C18:0, C18:3 n6, and C20:3 n6 showed an upward trend in phase III (from 5 to 13 DIM), and tended to decrease at the end of lactation. C18:3 n3 reached maximum values in phases III and IV, declining in the last phase, similarly to the findings by Barreto et al. [22].

C18:1 t11 showed higher values at the beginning of lactation, from postpartum to 4 DIM, then decreased and remained stable until the end of lactation. Other long-chain fatty acids (C18:1 t9, C18:2 n6t, and C20:3 n3) presented inconsistent trends.

The content of n3 PUFAs was higher in phases III and IV and showed the same trend as C18:3 n3, which is the main n3 fatty acid in equid milk. The n6/n3 ratio significantly increased in the V phase (Table 3). However, other authors found no significant variations in the n6/n3 ratio in equid milk [21,24].

## 4. Conclusions

This is the first study to report in detail the qualitative composition of mule milk. The mule milk was found to be very similar to horse and donkey milk in terms of the composition. The outcomes of this study should be considered alongside the limitations, since they were generated from only a single individual. However, they provide important basic information for understanding mule lactation. This study seems to support the hypothesis that the milk of taxonomically close species is nutritionally similar. The mule milk composition changed throughout lactation, probably following the growth requirements of the foal. 

## Figures and Tables

**Table 1:** Average mule milk chemical and fatty acid composition.

Parameter	Mean ± SD	Parameter	Mean ± SD	Parameter	Mean ± SD	Parameter	Mean ± SD
Dry matter (g 100 mL^−1^ milk)	10.84 ± 1.59	C4:0	0.06 ± 0.03	C18:1 c9	23.69 ± 2.48	C23:0	0.02 ± 0.01
Protein (g 100 mL^−1^ milk)	1.97 ± 0.55	C6:0	0.25 ± 0.10	C18:2 n6 t9,12	0.02 ± 0.02	C24:0	0.04 ± 0.02
Fat (g 100 mL^−1^ milk)	0.90 ± 0.75	C8:0	3.57 ± 0.61	C18:2 n6 c9,12	9.53 ± 1.35	C24:1	0.05 ± 0.02
Ash (g 100 mL^−1^ milk)	0.39 ± 0.13	C10:0	7.55 ± 1.87	C18:3 n3	7.80 ± 1.47		
Ca (mg L^−1^ milk)	942.79 ± 268.00	C11:0	0.02 ± 0.01	C18:3 n6	0.05 ± 0.04		
P (mg L^−1^ milk)	588.05 ± 184.41	C12:0	7.49 ± 1.54	CLA c9,t11	0.04 ± 0.02		
K (mg L^−1^ milk)	748.09 ± 260.48	C13:0	0.04 ± 0.01	C20:0	0.04 ± 0.01		
Mg (mg L^−1^ milk)	86.74 ± 184.41	C14:0	6.92 ± 1.10	C20:1 c11	0.38 ± 0.07		
Zn (mg L^−1^ milk)	2.31 ± 1.15	C14:1 c9	0.51 ± 0.12	C20:2	0.21 ± 0.09		
SFAs ^1^ (g 100 g^−1^ of fat)	50.41 ± 4.30	C15:0	0.37 ± 0.09	C20:3 n6	0.04 ± 0.01		
MUFAs ^2^ (g 100 g^−1^ of fat)	31.79 ± 3.45	C15:1 c10	0.17 ± 0.06	C20:3 n3	0.14 ± 0.16		
PUFAs ^3^ (g 100 g^−1^ of fat)	18.11 ± 1.82	C16:0	22.24 ± 2.14	C20:4 n6	0.03 ± 0.02		
SCFAs ^4^ (g 100 g^−1^ of fat)	11.57 ± 2.23	C16:1	0.60 ± 0.18	C20:5 n3	0.02 ± 0.02		
MCFAs ^5^ (g 100 g^−1^ of fat)	48.83 ± 3.12	C16:1 c9	5.78 ± 1.21	C21:0	0.003 ± 0.002		
LCFAs ^6^ (g 100 g^−1^ of fat)	44.00 ± 3.81	C17:0	0.33 ± 0.06	C22:0	0.15 ± 0.10		
UFA/SFA ^7^	0.99 ± 0.17	C17:1 c10	0.51 ± 0.07	C22:1	0.06 ± 0.03		
n3 (g 100 g^−1^ of fat)	8.09 ± 1.52	C18:0	1.51 ± 0.20	C22:2	0.006 ± 0.003		
n6 (g 100 g^−1^ of fat)	9.67 ± 1.35	C18:1 t9	0.02 ± 0.01	C22:5 n3	0.11 ± 0.02		
n6/n3	1.23 ± 0.37	C18:1-t11	0.01 ± 0.009	C22:6 n3	0.02 ± 0.01		

^1^ SFAs, saturated fatty acids; ^2^ MUFAs, monounsaturated fatty acids; ^3^ PUFAs, polyunsaturated fatty acids; ^4^ SCFAs, short-chain fatty acids, ≤C10; ^5^ MCFAs, medium-chain fatty acids, between C11 and C17; ^6^ LCFAs, long-chain fatty acids, ≥C18; ^7^ UFA/SFA, unsaturated/saturated fatty acid ratio.

**Table 2:** Gross and mineral composition of mule milk during lactation (least-squares means ± SE).

			Lactation Phase		
Parameter	I	II	III	IV	V
Dry matter (g 100 mL^−1^ milk)	12.35 ^A^ ± 0.38	13.32 ^A^ ± 0.47	12.44 ^A^ ± 0.40	10.28 ^B^ ± 0.23	9.33 ^C^ ± 0.23
Protein (g 100 mL^−1^ milk)	2.66 ^A^ ± 0.15	2.84 ^A^ ± 0.18	2.44 ^A^ ± 0.15	1.88 ^B^ ± 0.09	1.39 ^C^ ± 0.09
Fat (g 100 mL^−1^ milk)	1.87 ^A^ ± 0.23	1.90 ^A^ ± 0.28	1.85 ^A^ ± 0.23	0.39 ^B^ ± 0.14	0.29 ^B^ ± 0.08
Ash (g 100 mL^−1^ milk)	0.49 ^A^ ± 0.03	0.57 ^A^ ± 0.04	0.52 ^A^ ± 0.03	0.38 ^B^ ± 0.03	0.25 ^C^ ± 0.02
Ca (mg L^−1^ milk)	494.30 ^C^ ± 100.96	1159.18 ^AB^ ± 123.65	1208.99 ^A^ ± 100.96	912.55 ^B^ ± 61.82	1001.27 ^B^ ± 100.95
P (mg L^−1^ milk)	815.16 ^A^ ± 70.86	898.34 ^A^ ± 86.79	730.70 ^A^ ± 70.86	504.62 ^B^ ± 43.39	438.75 ^B^ ± 43.39
Mg (mg L^−1^ milk)	143.98 ^A^ ± 13.27	110.04 ^AB^ ± 16.26	109.98 ^AB^ ± 13.27	89.99 ^B^ ± 8.13	48.20 ^C^ ± 8.13
K (mg L^−1^ milk)	1136.62 ^A^ ± 116.54	1104.95 ^A^ ± 142.74	872.01 ^AB^ ± 116.54	683.65 ^BC^ ± 71.37	546.94 ^C^ ± 71.37
Zn (mg L^−1^ milk)	1.36 ± 0.73	2.47 ± 0.90	1.65 ± 0.73	2.14 ± 0.45	2.70 ± 0.73

^A, B, C^, means with a different superscript letter within a row differ significantly (*p* < 0.01). I, from 6 to 24 h postpartum; II, from 2 to 4 days in milk (DIM); III, from 5 to 14 DIM; IV, from 17 to 90 DIM; V, from 90 to 180 DIM.

**Table 3:** Fatty acid profile of mule milk during lactation (g 100 g^−1^ of fat) (least-square means ± SE).

			Lactation Phase		
Parameter	I	II	III	IV	V
C4:0	0.07 ^B^ ± 0.01	0.11 ^A^ ± 0.01	0.11 ^A^ ± 0.01	0.06 ^B^ ± 0.01	0.06 ^B^ ± 0.01
C6:0	0.08 ^C^ ± 0.03	0.19 ^BC^ ± 0.06	0.16 ^C^ ± 0.03	0.33 ^A^ ± 0.02	0.25 ^B^ ± 0.02
C8:0	3.17 ± 0.31	3.70 ± 0.53	3.53 ± 0.24	4.01 ± 0.17	3.18 ± 0.17
C10:0	9.12 ^A^ ± 0.66	9.77 ^A^ ± 0.66	7.98 ^AB^ ± 0.51	8.09 ^AB^ ± 0.36	6.50 ^B^ ± 0.38
C11:0	0.03 ± 0.01	0.03 ± 0.01	0.02 ± 0.01	0.02 ± 0.005	0.02 ± 0.005
C12:0	8.54 ± 0.83	9.05 ± 1.44	7.37 ± 0.65	7.45 ± 0.46	7.37 ± 0.48
C13:0	0.05 ± 0.01	0.06 ± 0.01	0.05 ± 0.005	0.04 ± 0.004	0.05 ± 0.004
C14:0	6.23 ± 0.65	7.58 ± 11.12	6.53 ± 0.50	6.72 ± 0.35	7.68 ± 0.37
C14:1 c9	0.47 ^b^ ± 0.07	0.52 ^ab^ ± 0.11	0.40 ^b^ ± 0.05	0.49 ^b^ ± 0.04	0.64 ^a^ ± 0.04
C15:0	0.24 ^C^ ± 0.02	0.30 ^BC^ ± 0.04	0.30 ^BC^ ± 0.02	0.34 ^B^ ± 0.01	0.49 ^A^ ± 0.01
C15:1 c10	0.10 ^C^ ± 0.02	0.14 ^BC^ ± 0.04	0.15 ^B^ ± 0.02	0.17 ^B^ ± 0.01	0.24 ^A^ ± 0.01
C16:0	19.64 ^B^ ± 0.82	22.48 ^AB^ ± 1.41	21.21 ^B^ ± 0.63	21.51 ^B^ ± 0.45	23.81 ^A^ ± 0.47
C16:1	0.81 ^a^ ± 0.08	0.63 ^ab^ ± 0.14	0.66 ^ab^ ± 0.06	0.63 ^a^ ± 0.04	0.45 ^b^ ± 0.05
C16:1 c9	6.11 ± 0.69	5.29 ± 1.20	5.68 ± 0.53	6.06 ± 0.38	5.42 ± 0.40
C17:0	0.27 ^C^ ± 0.02	0.35 ^AB^ ± 0.04	0.30 ^BC^ ± 0.02	0.30 ^BC^ ± 0.01	0.37 ^A^ ± 0.01
C17:1 c10	0.52 ± 0.04	0.54 ± 0.07	0.48 ± 0.03	0.47 ± 0.02	0.55 ± 0.02
C18:0	1.52 ^AB^ ± 0.08	1.55 ^AB^ ± 0.14	1.74 ^A^ ± 0.06	1.50 ^AB^ ± 0.04	1.31 ^B^ ± 0.05
C18:1 t9	0.02 ^ab^ ± 0.01	0.01 ^b^ ± 0.01	0.01 ^b^ ± 0.01	0.03 ^a^ ± 0.005	0.02 ^ab^ ± 0.005
C18:1 t11	0.02 ^a^ ± 0.004	0.02 ^a^ ± 0.005	0.01 ^b^ ± 0.003	0.01 ^b^ ± 0.002	0.01 ^b^ ± 0.002
C18:1 c9	24.54 ± 1.52	23.22 ± 1.86	22.76 ± 1.18	23.27 ± 0.83	22.94 ± 0.83
C18:2 n6(t9,12)	0.05 ^a^ ± 0.01	0.03 ^ab^ ± 0.01	0.01 ^b^ ± 0.008	0.04 ^a^ ± 0.006	0.02 ^b^ ± 0.006
C18:2 n6(c9,12)	9.36 ± 0.66	9.97 ± 0.81	9.89 ± 0.51	8.57 ± 0.36	10.34 ± 0.38
C18:3 n3(9,12,15)	7.72 ^ab^ ± 0.71	7.34 ^ab^ ± 0.87	9.02 ^a^ ± 0.55	8.39 ^a^ ± 0.39	6.33 ^b^ ± 0.39
C18:3 n6(6,9,12)	0.06 ^B^ ± 0.01	0.10 ^AB^ ± 0.02	0.11 ^A^ ± 0.01	0.03 ^C^ ± 0.01	0.01 ^D^ ± 0.01
CLA c9,t11	0.02 ± 0.01	0.01 ± 0.01	0.03 ± 0.008	0.03 ± 0.006	0.04 ± 0.006
C20:0	0.03 ± 0.008	0.03 ± 0.01	0.04 ± 0.006	0.04 ± 0.005	0.04 ± 0.005
C20:1 c11	0.47 ± 0.04	0.37 ± 0.04	0.38 ± 0.03	0.36 ± 0.02	0.36 ± 0.02
C20:2	0.06 ^B^ ± 0.03	0.16 ^AB^ ± 0.04	0.27 ^A^ ± 0.03	0.25 ^A^ ± 0.02	0.22 ^A^ ± 0.02
C20:3 n6(8,11,14)	0.04 ^AB^ ± 0.006	0.05 ^A^ ± 0.007	0.06 ^A^ ± 0.005	0.03 ^B^ ± 0.003	0.03 ^B^ ± 0.003
C20:3 n3(11,14,17)	0.11 ^B^ ± 0.07	0.01 ^B^ ± 0.01	0.01 ^B^ ± 0.01	0.33 ^A^ ± 0.01	0.10 ^B^ ± 0.01
C20:4 n6	0.02 ± 0.01	0.02 ± 0.01	0.02 ± 0.01	0.06 ± 0.01	0.02 ± 0.01
C20:5 n3	0.03 ± 0.01	0.01 ± 0.01	0.02 ± 0.01	0.02 ± 0.01	0.03 ± 0.01
C21:0	0.003 ± 0.002	0.004 ± 0.002	0.002 ± 0.001	0.002 ± 0.001	0.003 ± 0.001
C22:0	0.23 ± 0.07	0.24 ± 0.08	0.22 ± 0.05	0.14 ± 0.04	0.24 ± 0.04
C22:1	0.05 ± 0.02	0.03 ± 0.02	0.08 ± 0.01	0.07 ± 0.009	0.06 ± 0.009
C22:2	0.005 ± 0.002	0.002 ± 0.001	0.003 ± 0.001	0.009 ± 0.002	0.005 ± 0.002
C22:5 n3	0.10 ± 0.01	0.11 ± 0.01	0.12 ± 0.01	0.10 ± 0.006	0.09 ± 0.006
C22:6 n3	0.02 ± 0.01	0.02 ± 0.01	0.03 ± 0.005	0.03 ± 0.004	0.02 ± 0.004
C23:0	0.01 ± 0.006	0.02 ± 0.007	0.02 ± 0.004	0.02 ± 0.003	0.01 ± 0.003
C24:0	0.02 ± 0.01	0.02 ± 0.01	0.04 ± 0.01	0.06 ± 0.01	0.03 ± 0.01
C24:1	0.03 ± 0.01	0.03 ± 0.01	0.05 ± 0.01	0.05 ± 0.01	0.05 ± 0.01
SFAs ^1^	49.24 ± 2.64	55.35 ± 4.57	49.82 ± 2.04	50.50 ± 1.44	52.01 ± 1.61
MUFAs ^2^	33.14 ± 2.03	30.80 ± 3.51	30.63 ± 1.57	31.61 ± 1.11	30.75 ± 1.24
PUFAs ^3^	17.60 ± 1.06	17.82 ± 1.84	19.55 ± 0.82	17.89 ± 0.58	17.23 ± 0.65
SCFAs ^4^	12.44 ± 0.98	14.14 ± 1.70	12.03 ± 0.76	12.47 ± 0.54	10.44 ± 0.60
MCFAs ^5^	43.00 ± 1.57	46.98 ± 2.73	43.13 ± 1.22	44.20 ± 0.86	47.28 ± 0.96
LCFAs ^6^	44.56 ± 2.15	43.34 ± 3.72	44.84 ± 1.66	43.33 ± 1.18	42.28 ± 1.32
UFA/SFA ^7^	1.06 ± 0.11	0.96 ± 0.18	1.01 ± 0.10	0.99 ± 0.06	0.94 ± 0.06
n3	7.99 ^ab^ ± 0.65	7.50 ^ab^ ± 1.13	9.19 ^a^ ± 0.50	8.88 ^a^ ± 0.36	6.56 ^b^ ± 0.40
n6	9.54 ± 0.69	10.15 ± 1.21	10.06 ± 0.54	8.73 ± 0.38	10.41 ± 0.43
n6/n3	1.20 ^B^ ± 0.15	1.43 ^AB^ ± 0.27	1.11 ^B^ ± 0.11	0.99 ^B^ ± 0.08	1.66 ^A^ ± 0.09

^a, b,^, means with a different superscript letter within a row differ significantly (*p* < 0.05); ^A, B, C, D^, means with a different superscript letter within a row differ significantly (*p* < 0.01); ^1^ SFAs, saturated fatty acids; ^2^ MUFAs, monounsaturated fatty acids; ^3^ PUFAs, polyunsaturated fatty acids; ^4^ SCFAs, short-chain fatty acids, ≤C10; ^5^ MCFAs, medium-chain fatty acids, between C11 and C17; ^6^ LCFAs, long-chain fatty acids, ≥C18; ^7^ UFA/SFA, unsaturated/saturated fatty acid ratio. I, from 6 to 24 h postpartum; II, from 2 to 4 days in milk (DIM); III, from 5 to 14 DIM; IV, from 17 to 90 DIM; V, from 90 to 180 DIM.

## Data Availability

The datasets generated during and/or analysed during the current study are available from the corresponding author on reasonable request.

## References

[1] Fanelli D., Moroni R., Bocci C., Camillo F., Rota A., Panzani D. (2023). Interspecific and Intraspecific Artificial Insemination in Domestic Equids. Animals.

[2] Allen W.R., Short R.V. (1997). Interspecific and Extraspecific Pregnancies in Equids: Anything Goes. J. Hered..

[3] Proops L., Burden F., Osthaus B. (2009). Mule cognition: A case of hybrid vigour?. Anim. Cogn..

[4] Carluccio A., Bucci R., Fusi J., Robbe D., Veronesi M.C. (2020). Effect of age and of reproductive status on reproductive indices in horse mares carrying mule pregnancies. Heliyon.

[5] Camillo F., Vannozzi I., Rota A., Luzio B., Romagnoli S., Aria G., Allen W.R. (2003). Successful Non-Surgical Transfer of Horse Embryos to Mule Recipients. Reprod. Domest. Anim..

[6] Stout T.A.E. (2006). Equine embryo transfer: Review of developing potential. Equine Vet. J..

[7] Summers P.M., Shephard A.M., Hodges J.K. (1987). Successful transfer of the embryos of Przewalski’s horses (*Equus przewalskii*) and Grant’s zebra (*E. burchelli*) to domestic mares (*E. caballus*). J. Reprod. Fertil..

[8] González S.M., Gomes R.G., Souza A.K., Silva C.B., Silva-Santos K.C., Seneda M.M. (2015). Evidences of regular estrous cycles in mules and successful use of these animals as recipients for donkey embryos. J. Equine Vet. Sci..

[9] Bottrel M., Fortes T., Ortiz I., Hidalgo M., Dorado J. (2018). Short communication: Establishment and maintenance of donkey-in-mule pregnancy after embryo transfer in a non-cycling mule treated with oestradiol benzoate and long-acting progesterone. Span. J. Agric. Res..

[10] Uniacke-Lowe T., Fox P.F., Fuquay J.W. (2011). Milk|Equid Milk. Encyclopedia of Dairy Sciences.

[11] Oftedal O.T., Jenness R. (1988). Interspecies variation in milk composition among horses, zebras and asses (Perissodactyla: Equidae). J. Dairy Res..

[12] Association of Official Analytical Chemists (AOAC) (2000). Official Methods of Analysis.

[13] Altomonte I., Conte G., Serra A., Mele M., Cannizzo L., Salari F., Martini M. (2019). Nutritional characteristics and volatile components of sheep milk products during two grazing seasons. Small Rumin. Res..

[14] JMP (2002). User’s Guide.

[15] Schryver H.F., Oftedal O.T., Williams J., Cymbaluk N.F., Antczak D., Hintz H.F. (1986). A comparison of the mineral composition of milk of domestic and captive wild equids (*Equus przewalski*, *E. zebra*, *E. burchelli*, *E. caballus*, *E. assinus*). Comp. Biochem. Physiol. Mol..

[16] Altomonte I., Salari F., Licitra R., Martini M. (2019). Donkey and human milk: Insights into their compositional similarities. Int. Dairy J..

[17] Musaev A., Sadykova S., Anambayeva A., Saizhanova M., Balkanay G., Kolbaev M. (2021). Mare’s Milk: Composition, Properties, and Application in Medicine. Arch. Razi Inst..

[18] Becvarova I., Buechner-Maxwell V. (2012). Feeding the foal for immediate and long-term health. Equine Vet. J..

[19] Martemucci G., D’Alessandro A.G. (2012). Fat content, energy value and fatty acid profile of donkey milk during lactation and implications for human nutrition. Lipids Health Dis..

[20] Li M., Liu Y., Li Q., Yang M., Pi Y., Yang N., Zheng Y., Yue X. (2020). Comparative exploration of free fatty acids in donkey colostrum and mature milk based on a metabolomics approach. J. Dairy Sci..

[21] Pikul J., Wó Jtowski J., Dankó R., Kuczyn’ska B., Kuczyn´ska K., Łojek J. (2008). Fat content and fatty acids profile of colostrum and milk of primitive Konik horses (*Equus caballus gmelini* Ant.) during six months of lactation. J. Dairy Res..

[22] Barreto Í.M.L.G., Urbano S.A., Oliveira C.A.A., Macêdo C.S., Borba L.H.F., Chags B.M.E., Rangel A.H.N. (2020). Chemical composition and lipid profile of mare colostrum and milk of the quarter horse breed. PLoS ONE.

[23] Martini M., Licitra R., Altomonte I., Salari F. (2020). Quality of donkey mammary secretion during the first ten days of lactation. Int. Dairy J..

[24] Martini M., Altomonte I., Manica E., Salari F. (2015). Changes in donkey milk lipids in relation to season and lactation. J. Food Compos. Anal..

[25] Innis S.M. (2016). Palmitic Acid in Early Human Development. Crit. Rev. Food Sci. Nutr..

[26] Salimei E., Fantuz F. (2012). Equid milk for human consumption. Int. Dairy J..

[27] D’Alessandro A.G., Martemucci G. (2012). Lactation curve and effects of milking regimen on milk yield and quality, and udder health in Martina Franca jennies (*Equus asinus*). J. Anim. Sci..

[28] Markiewicz-Kęszycka M., Wójtowski J., Gryżak-Runowska G., Kuczyńska B., Puppel K., Krzyżewski J. (2015). Influence of stage of lactation and year season on composition of mares’ colostrum and milk and method and time of storage on vitamin C content in mares’ milk. J. Sci. Food Agric..

[29] Kouba J.M., Burns T.A., Webel S.K. (2019). Effect of dietary supplementation with long-chain n-3 fatty acids during late gestation and early lactation on mare and foal plasma fatty acid composition, milk fatty acid composition, and mare reproductive variables. Anim. Reprod. Sci..

[30] Salari F., Ciampolini R., Mariti C., Millanta F., Altomonte I., Licitra R., Auzino B., Bibbiani C., Giuliotti L., Papini R.A. (2019). A multi-approach study of the performance of dairy donkey during lactation: Preliminary results. Ital. J. Anim. Sci..

[31] Fantuz F., Ferraro S., Todini L., Piloni R., Mariani P., Salimei E. (2012). Donkey milk concentration of calcium, phosphorus, potassium, sodium and magnesium. Int. Dairy J..

[32] Martini M., Altomonte I., Salari F., Caroli A.M. (2014). Short communication: Monitoring nutritional quality of Amiata donkey milk: Effects of lactation and productive season. J. Dairy Sci..

[33] Salari F., Altomonte I., Boselli C., Martini M. (2023). Amiata donkey body conformation, udder characteristics, and their relationship with milk yield and quality. Can. J. Anim. Sci..

[34] Salimei E., Fantuz F., Coppola R., Chiofalo B., Polidori P., Varisco G. (2004). Composition and characteristics of ass’s milk. Anim. Res..

[35] Guo H.Y., Pang K., Zhang X.Y., Zhao L., Che S.W., Dong M.L., Ren F.Z. (2007). Composition, physiochemical properties, nitrogen fraction distribution and amino acid profile of donkey milk. J. Dairy Sci..

[36] Summer A., Sabbioni P., Formaggioni P., Mariani P. (2004). Trend in ash and mineral element content of milk from Haflinger nursing mares throughout six lactation months. Livest. Prod. Sci..

